# Are Turkish pharmaceutical pricing strategies an access barrier to oncology medicines for Türkiye?

**DOI:** 10.3389/fphar.2024.1364341

**Published:** 2024-05-10

**Authors:** Elif Hilal Vural, Tolga Kaskati, İsmail Mert Vural, Mustafa Asım Özalp, Bülent Gümüşel

**Affiliations:** ^1^ Medical Pharmacology Department, Faculty of Medicine, Lokman Hekim University, Ankara, Türkiye; ^2^ BYS Grup AŞ, Ankara, Türkiye; ^3^ Department of Pharmacology, Gulhane Faculty of Pharmacy, University of Health Sciences Türkiye, Ankara, Türkiye; ^4^ Department of Actuarial Sciences, Faculty of Science, Hacettepe University, Ankara, Türkiye; ^5^ Pharmacology Department, Faculty of Pharmacy, Lokman Hekim University, Ankara, Türkiye

**Keywords:** pharmacoeconomy, medicine pricing, oncology, medicine access, barriers

## Abstract

**Objectives:** Cancer diagnosis is increasing day by day all over the world. Deaths due to cancer are among the most common causes of death. Access to cancer drugs is a priority of health policies. The aim of this study is to evaluate access to cancer drugs through drug box sales data by modeling population growth, cancer incidence, and Fixed Euro Exchange (FEE) rate parameters used in drug pricing in Türkiye.

**Methods:** Access to cancer drugs was evaluated by drug box sales figures obtained from IQVIA. Box sales data were classified according to diagnosis codes (ICD-10), reference, or generic status. Consumption of cancer drugs was examined over time with panel regression analysis, taking into account variables of population growth, cancer incidence, and the FEE rate in drug pricing in Türkiye.

**Results:** The incidence of cancer in Türkiye was 215.1 in 2010 and 223.1 (per hundred thousand) in 2017. Whereas there was a 127.02% increase in the real euro exchange rate, there was an 89.6% increase in the FEE rate. With the regression approach, there is a negative relationship between the real and fixed exchange rate difference (RFED) and reference and generic drug consumption data. Medicine access is affected depending on diagnosis codes at different levels. Colorectal cancer medicine sales had negative correlations for each variable, namely, exchange rate, population growth, and cancer incidence. On the contrary, there was a positive correlation between non-small-cell lung cancer and relevant variables. Innovative medicine groups such as monoclonal antibodies and protein kinase inhibitor consumption showed a negative correlation.

**Conclusion:** According to our results, pricing strategy may be an access barrier for oncology medicines in Türkiye. It should be reviewing the pricing policy that is beneficial for oncology medicine access in Türkiye.

## Introduction

Cancer is a significant noncommunicable health problem worldwide. According to the GLOBOCAN 2020 records, approximately 19 million new cancer cases and 10 million cancer-related deaths occurred all over the world (Sung et al., 2021). Despite the fact that cancer is the second leading cause of early death after cardiovascular-related death in the 20th century, it is projected to rank first in the near future (Bray et al., 2021). The most prevalent cancer types in both sexes are lung (17.1%), breast (10.5%), colorectal (9.0%), prostate (7.2%), and thyroid (6.4%) cancers, according to GLOBOCAN 2022 statistics in Türkiye (The Global Cancer Observatory GLOBOCAN 2020, 2024). The Cancer Control Program was established to prevent cancer development and cancer-related deaths, with available analysis of the situation, determination of priorities, and determination of appropriate strategies in Türkiye. There have been important developments such as the Turkish Cancer Control and Research Institute establishment in 1947 or the introduction of Cancer Early Diagnosis, Screening, and Training Centers in 1995 (Sağlık Bakanlığı, 2021). Despite all developments, cancer is still diagnosed at advanced stages in Türkiye. According to the National Breast Cancer Registry Program of the Turkish Federation of Breast Diseases Societies data, 71.5% of breast cancer patients had Stage II or higher cancer at diagnosis (Özmen et al., 2019). Increased screening programs, early diagnosis centers, and epidemiological, genetic, and molecular research are still needed (Özmen et al., 2016).

Innovative cancer treatment agents are the subject of various health economic research studies due to their budget impact. Previous studies reported that oncology pharmaceutical expenses constitute approximately 10%–20% of cancer-related healthcare costs (Jönsson and Wilking, 2007). Globally, cancer medicine spending was US$ 196 billion in 2022, and breast cancer, non-small-cell lung cancer, prostate cancer, multiple myeloma, and kidney cancer pharmaceuticals accounted for more than half of cancer medicine sales between 2018 and 2022 (IQVIA Institute Report, 2023). The economic burden of cancer reported in studies conducted in Europe was €18.8 billion for lung cancer, which was the highest economic cost among other cancers in 2009. Only 4% of this cost was pharmaceutical expenditures (Luengo-Fernandez et al., 2013). Whereas in Europe cancer medicine expenditures were €10 billion in 2005, this cost reached €32 billion, which was 31% of total spending in 2018 (Hofmarcher et al., 2020). Cicin et al. have reported that the lung cancer annual cost for Türkiye from a payer perspective is almost 498 million euros, and 26% of this cost is medical treatment (Cicin et al., 2021).

After pharmaceutical products were licensed, many market barrier factors, such as national price settings and/or negotiations, and reimbursement decisions affected the patient’s access to cancer pharmaceuticals (Jönsson and Wilking, 2007). Spending control, efficiency evaluations, and access criteria in the pharmaceutical market are important political interventions for population health status (Maynard and Bloor, 2003).

The purpose of the health transition program in Türkiye launched in 2003 was to improve the quality and management of health services, and to provide financial sustainability for health (Saglik, 2003). This program increases access to medicine and health services and improves health outcomes and patient satisfaction (Ökem and Çakar, 2015). Whereas the incidence of cancer in Türkiye was 189.6 (per hundred thousand) in 2004 Türkiye Cancer Statistic Yearbook 2009 (Turkiye Kanser Istatistikleri Yillıgi, 2014), it was 223.1 (per hundred thousand) in 2017 according to Türkiye Cancer Statistic Yearbook 2018 (Turkiye Kanser Istatistikleri Yillıgi, 2022). In addition to the increasing population and increasing cancer diagnosis/incidence, health insurance is widely accessible in Türkiye. Population coverage in the general health insurance system had reached 99% in Türkiye Pharmaceutical, and related expenditures constituted 34% of total health expenditures in 2021 (SGK, 2023). The Turkish medical pricing system is under the control of the Republic of Türkiye Ministry of Health, and the pricing system was changed with the health transition program. External pricing systems and fixed-rate currencies are both main determinants of the medicine pricing mechanism in Türkiye (Saglik, 2003). Fixed-rate currency is the Euro exchange value determined by the Price Evaluation Commission (FDK) and is valid to be used in the calculation of all medicine prices until their redetermination in Türkiye. This application was started in 2004. The first fixed exchange rate was determined to be equal to the real Euro/TL exchange rate (1€ = 1.6317 TL (Turkish Lira)). After 5 years, the fixed exchange rate used to calculate medicine prices was 1.9595 TL, whereas the real euro exchange rate was 2.1181. The Price Evaluation Commission raised the fixed exchange rate to 14.0387 TL, whereas the real exchange rate was 29.9829 TL in July 2023 (TISD Turkiye Ilac Sanayi Dernegi, 2024).

As a result, the gap increased in the following years between the fixed euro exchange (FEE) rate and the real exchange rate in Türkiye. Based on this observation, the purpose of our study was to determine whether access to cancer medicines in Türkiye is affected by the difference between the fixed exchange rate and the real exchange rate with the panel regression analysis in the early years when the difference began to be witnessed.

## Material and methods

### Data sources and properties

This study analyzed the consumption of oncology medicines between 2010 and 2017. Data used have been obtained from IQVIA Health for this scientific study. IQVIA Health is an international pharmaceutical consulting company that collects sales and price data at the level within the pharmaceutical market supply and distribution chain. These data also include corrections of returns from the pharmacy to the warehouse. The total of retail and hospital box sales data was the accepted value for the consumption of medication or access to medicine in this study. The data include monthly box sales by active ingredients and pharmaceuticals classified by the Anatomical Therapeutic Chemical (ATC) classification system. It includes box sales of the “L01-Antineoplastic Agents” group between 2003 and 2022. The data are anonymized and do not contain any patient/personal or prescription information.

All data were grouped into manufactured or imported and original or generic. Active substances were clustered according to their approved indications by the Ministry of Health to evaluate medicines used in certain cancer diagnoses. Cancer incidence data between 2004 and 2017 were obtained from the Ministry of Health Türkiye Cancer Statistic Yearbook 2009, 2018, and 2014 (Turkiye Kanser Istatistikleri Yilligi, 2014; Turkiye Kanser Istatistikleri Yilligi, 2017; Turkiye Kanser Istatistikleri Yilligi, 2022). The real Euro/TL exchange rate was obtained from the Central Bank of the Republic of Türkiye. The fixed exchange rates used to determine medicine prices were obtained from the Turkish Ministry of Health.

As there was no difference between the real exchange rate and the fixed exchange rate between 2002 and 2009, data obtained before 2010 were excluded from the evaluation. As oncology medicines have been obtained with public procurement since 2018 through the Health Market Application, which is an electronic tender system, data were not included in the analysis from 2018 and later.

All analyses were conducted using the statistical software R program.

### Data modeling

Box sales data for cancer medicines were examined over time with the panel regression analysis, taking into account variables of population growth, cancer incidence, and the FEE rate in medicine pricing in Türkiye. The normality of the distribution of data was evaluated using the Shapiro–Wilk test.

### Regression analysis

Regression analysis is a powerful statistical tool used in various fields to examine relationships between variables. This section provides an overview of linear regression and panel regression methods, outlining their principles, equations, and applications in data analysis.

Linear regression is a fundamental statistical method used to model the relationship between one or more independent variables (denoted as X) and a dependent variable (usually represented by the letter Y). This method assumes a linear relationship, meaning that changes in the independent variables correspond to consistent variations in the dependent variable. The equation for simple linear regression, involving one independent variable and one dependent variable, can be expressed as follows:

Y=βX+ϵ,

where X is the independent variable (the variable we use to make predictions) and Y is the dependent variable (the variable we wish to forecast). The change in Y for a one-unit change in X is represented by the coefficient vector, β. The error term, represented by ε, measures the discrepancy between the value of Y as observed and the value anticipated by the model (Darlington and Hayes, 2017).

Panel regression, also known as longitudinal data analysis or fixed effects regression, is employed when data are collected for multiple individuals or entities (cross-sectional) across various time periods (time series). It extends the principles of linear regression by handling both time series and cross-sectional data simultaneously. This approach allows for the analysis of individual-specific effects (fixed effects) and time-specific effects (time effects), enabling a comprehensive understanding of how individuals or entities develop over time while controlling for specific factors (Kiu et al., 2019; Nie et al., 2020; Cheng et al., 2021).

The model specification is considered as the following panel data model with two effects:

$$y_{it}=\beta X_{it}+\alpha_{i}+\gamma_{t}+u_{it},$$

where $y_{it}$ is the dependent variable (sales boxes) for month i at year t, β represents the coefficients of the explanatory variables, $X_{it},\alpha_{i}$ represents the month-specific effect (*i* = 1 … 12), $\gamma_{t}$ represents the time-specific effect (t = 2010, … 2017), and $u_{it}$ is the error term. These components allow for a comprehensive control of unobserved factors across both dimensions (Wooldridge, 2010).

The relationship between consumed oncology medicine amount and population or cancer incidence or exchange rate difference was evaluated by the coefficients of regression analysis. We also developed a model with the panel regression approach according to the 3rd level of ATC groups. Models were built for each ATC group’s box sales data. Sales data impact was analyzed with each parameter population, cancer incidence, and exchange rate difference.

The International Statistical Classification of Diseases and Related Health Problems (ICD-10) codes were also used in this study to categorize groups of active substances. The possible effects caused by access barriers in certain diagnostic groups in terms of ICD-10 codes were also assessed. Therefore, unlike the previous analysis group, an active substance was included in the analysis in more than one diagnosis code. While making this classification, the approved indications of the products included in the summary product characteristic (SmPC) have been considered. We reported the final regression model results in terms of the regression coefficients, standard errors, and *p*-values.

## Results

### Epidemiological and pharmacoepidemiological data

It was observed that cancer incidence change was 3.72% and population growth was 9.61% between 2010 and 2017 (Table 1).

**TABLE 1:** Population, cancer incidence, and cancer incidence percentage changes in Türkiye between 2010 and 2017.

	2010	2011	2012	2013	2014	2015	2016	2017
Population (n)	73,722,988	74,724,269	75,627,384	76,667,864	77,695,904	78,741,053	79,814,871	80,810,525
Population percentage change from the prior year (%)		1.36	1.21	1.38	1.34	1.35	1.36	1.25
Cancer incidence	215.1	228.6	233	227.2	210.2	212.6	221.6	223.1
(rate per 100.000)								
Cancer incidence percentage change from the prior year (%)		6.28	1.92	−2.49	−7.48	1.14	4.23	0.68

**FIGURE 1:**
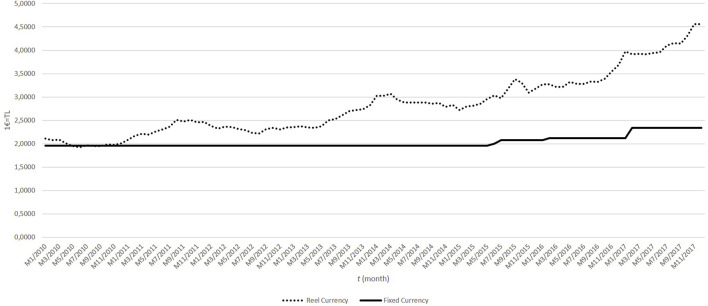
Fixed and real currency (1€ = TL) between 2010 and 2017 in Türkiye.

Whereas the fixed exchange rate that is used in pricing medicine in Türkiye changed by 89.60%, the real exchange rate changed by 127.02% between 2010 and 2017 (Figure 1).

To illustrate the variety of products available in the Turkish oncology market, we first determined the numbers of medicinal products and active substances in the pertinent years. Medicinal product numbers identify the number of formulations of the same active substance or products from different suppliers. The number of active substances and their medicinal products (in oncology treatment) between the years investigated is presented in Table 2.

**TABLE 2:** Medicinal products and active substances in Türkiye between 2010 and 2017.

	2010	2011	2012	2013	2014	2015	2016	2017
Active substances (n)	59	59	59	63	64	66	80	88
Medicinal product (n)	217	242	253	272	262	282	311	325

### General trends

**FIGURE 2:**
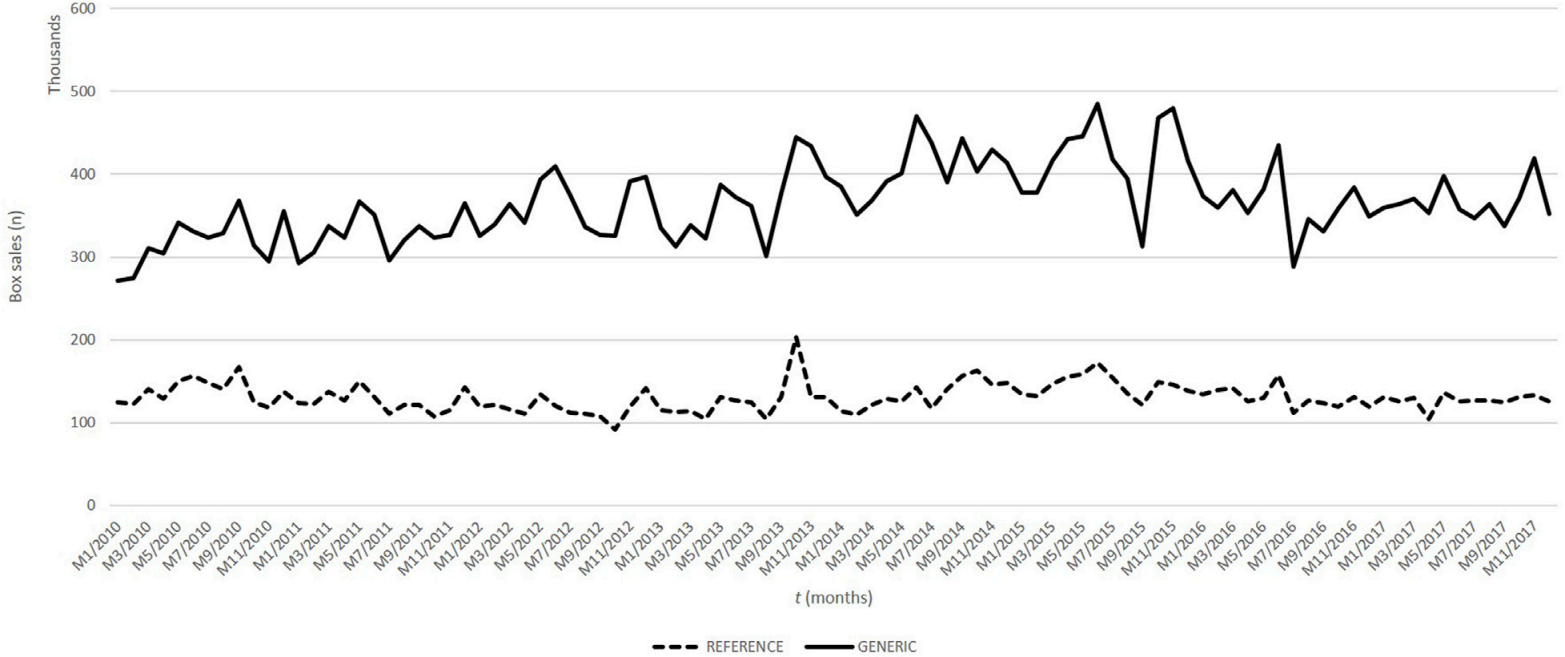
Oncology medicine box sales between 2010 and 2017 in Türkiye.

A general trend assessment was made using the Spearman correlation test to evaluate the consumption of oncology medicine box sales between 2010 and 2017 (Figure 2). We found a negative 57% correlation between total consumption of oncology medicines and the logarithm of the exchange rate difference at the 95% confidence level (*p*-value < 0.05).

### Panel data

There was a positive 81% relationship between the annual total number of oncology medicine boxes and the population at a 95% confidence level. However, we found that there was a 52% negative relationship between the monthly total oncology medicine sales and cancer incidence at a 95% confidence level between 2010 and 2017.

We also evaluate original and generic oncology medicine consumption. A model was built for the original medicinal consumption using the linear regression approach. The moderators accounted for multiple R2 = 0.9875 of 98% of the total explanatory capacity of the model. We found a negative relationship between the logarithm of the exchange rate difference and original oncology medicine consumption at a 99% confidence level. There was a positive relationship between population increase and original oncology medicine consumption at a 99% confidence level. We could not find a relationship between cancer incidence and original oncology medicine consumption.

When the model was developed in terms of generic oncology medicine consumption, a negative relationship was observed for all three evaluated parameters, that is, population increase, cancer incidence, or logarithm of exchange rate difference with a 99% confidence level (R^2^ = 0.9884, *p*-value: <0.05).

Alkylating agents (L01A), antimetabolites (L01B) group, plant-based neoplastic (L01C) group, antibiotic-based antineoplastics (L01D) group, platinum antineoplastic group (L01F), monoclonal antibodies antineoplastic group (L01G), protein kinase inhibitors antineoplastics (L01H), and other antineoplastics (L01X) panel regression analyses’ results are shown in Table 3.

**TABLE 3:** Panel regression analysis: impact of exchange rate difference, population rate, and cancer incidence rate on drug box sales by ATC groups.

ATC groups	Exchange rate difference (regression coefficient)		Population (regression coefficient)		Cancer incidence (regression coefficient)		R2
Alkylating agents (L01A)	−7,108.9**	(2,302.3)	6,477.5	(11,605)	−490.8*	(279)	0.92
Antimetabolites (L01B)	−176.1	(3,003.5)	−5,398.9**	(15,139.5)	−1,252.8**	(364.5)	0.98
Plant-based antineoplastics (L01C)	6,101.6**	(1867.5)	−29342**	(9,413.3)	−845.4*	(226.6)	0.99
Antibiotic-based antineoplastics (L01D)	8,282.4**	(1795.6)	16,353.6	(9,051.3)	−788.3*	(217.9)	0.96
Platinum antineoplastics (L01F)	8,136.9***	(1,136.1)	−32835***	(5,726.7)	−5,384.4	(137.9)	0.98
Monoclonal antibodies (L01G)	−225618.8***	(2097.7)	−3,995.7***	(10,575)	−239.5	(254.6)	0.96
Protein kinase inhibitors (L01H)	−5,585.1***	(268.9)	−918.1	(1,355.6)	23.5	(32.6)	0.98
All other group antineoplastics (L01X)	−4,169.6***	(448.2)	670.1	(2,259.3)	−30.85	(54.4)	0.97

Standard errors are in parentheses; *p < 0.05, **p < 0.01, ***p < 0.001.

Alkylating agents, monoclonal antibodies, protein kinase inhibitors, and other antineoplastics had negative relationships between exchange rate differences and box sales, but plant-based antineoplastics, antibiotic-based antineoplastics, and the platinum antineoplastic group had significantly positive correlations.

Antimetabolites, plant-based antineoplastics, platinum antineoplastics, and monoclonal antibodies showed negative relationships between population growth and box sales. Alkylating agents, antimetabolites, plant-based antineoplastics, and antibiotic-based antineoplastics had negative relationships between cancer incidence and box sales.

We could not build a model with proteasome inhibitor antineoplastics (L01J), Lidomide antineoplastics (L01K), and PARP inhibitor antineoplastics (L01L) data because their sales data did not show a trend. Their results were not shown.

Non-small-cell lung cancer, breast cancer, lymphoma, multiple myeloma, myeloid leukemia (acute-chronic), and colon and rectum cancer medicines were classified according to licensed indications. Many cancer diagnoses had a negative relationship between exchange rate differences and box sales without non-small-cell lung cancer and breast cancer. The majority of cancer diagnoses showed a positive relationship between population increase and box sales, but colorectal cancer had a negative relationship. Only non-small-cell lung cancer diagnosis box sales and cancer incidence had a positive relationship. The outcomes of the panel regression analysis for drug sales, focusing on specific cancer types, are presented in Table 4, incorporating exchange rate differences, population rates, and cancer incidence rates as key variables. Evaluation of R^2^ values in the final column reveals that the models exhibit significance, with explanation rates exceeding 80%.

**TABLE 4:** Panel regression analysis: impact of exchange rate difference, population rate, and cancer incidence rate on drug box sales by diagnosis code.

Box sales (monthly)	Exchange rate difference (regression coefficient)		Population (regression coefficient)		Cancer incidence (regression coefficient)		R^2^
Colorectal cancer (C18-20)	−9,617.2^***^	(1,523.4)	−573.2^***^	(106.5)	−30199.7^**^	(8,481.5)	0.99
Non-small-cell lung cancer (C34)	1,160.7^*^	(676.74)	1,495.8^***^	(237.4)	15,746.4^*^	(6,545.6)	0.85
Malign melanoma (C43)	−381.5^**^	(115.1)	16.1^**^	(4.8)	−1,215.4^*^	(726.96)	0.99
Breast cancer (C50)	1,305.6	(2,259.4)	−11495.9	(14,274.1)	195.01^*^	(95.68)	0.98
Multiple myeloma (C90)	−902.01^***^	(110.87)	1830^***^	(16.58)	−64.2^***^	(79.39)	0.96
Acute and chronic leukemia (C91-92-94)	−3,704.7^*^	(1801.4)	17,677.5^***^	(2,915)	−1,010.8^*^	(486.9)	0.99

Standard errors are in parentheses; *p < 0.05, **p < 0.01, ***p < 0.001.

For colorectal cancer, all three explanatory variables exhibit statistical significance within the model. Furthermore, an increase in these variables correlates with a reduction in drug box sales. Similarly, for non-small-cell lung cancer, all three explanatory variables are statistically significant at a 99% confidence level, indicating that an increase in these variables is associated with an augmentation in drug box sales. Moreover, non-small-cell lung cancer demonstrates statistical significance at a 95% confidence level for all three explanatory variables, with increases in these variables corresponding to heightened drug box sales.

Malignant melanoma exhibits statistical significance for all three explanatory variables at a 90% confidence level. Notably, an increase in the exchange rate and cancer incidence rate is linked to a decrease in drug box sales, whereas an increase in the population rate corresponds to an increase in sales.

Breast cancer, on the other hand, displays significance solely for the cancer incidence rate at a 90% confidence level. An escalation in the cancer incidence rate is associated with an increase in the number of medicine boxes sold.

In the case of multiple myeloma, all three explanatory variables attain statistical significance at a 99% confidence level within the model. The exchange rate difference and an increase in cancer incidence rate are linked to a reduction in drug box sales, whereas an increase in the population growth rate is associated with an increase in sales.

No relationship was found between the box sales data for the diagnosis of lymphoma and any of the parameters examined.

## Discussion

In this study, medicine access was evaluated through box sales data. Medicine access should be evaluated within the context of each country’s conditions, such as the healthcare system and the country’s income. Fundtytus et al. have reported that country income is one of the main factors that affect access to essential cancer medicines. Each country uses different mechanisms for medical pricing, considering their national priorities (Fundytus et al., 2021). Many European countries use direct price controls, international price comparisons, and/or reference pricing for determining pharmaceutical prices (Mossialos et al., 2004). In Türkiye, many regulations, including the reference price system, occurred with the Health Transformation Program started in 2003 for more equitable access to health for the whole society (Atun et al., 2013). One of the important components of the reference price system is the fixed euro exchange rate. After 2010, the gap between the fixed euro exchange rate and the real exchange rate increased year by year in Türkiye. It has been shown in this study that the increase in the difference between the fixed euro exchange rate and the real exchange rate affects access to oncology medicines in Türkiye. Similar to the results of our study, it was reported that strict price control and reimbursement mechanisms also lead to barriers to access to medicine in various countries, such as India and Poland. In that study, it was also mentioned that there are different barriers to access to oncology medicines, such as the high price of medicines and strict reimbursement criteria that limit diagnostic capacity, according to the opinions of six oncologists in different regions of the world. Moreover, each country has different barriers from each other (Barrios et al., 2023).

According to our results, the number of active substances increased from 59 to 88 in Türkiye. There is no country example where the number of products on the market can be compared head-to-head in the years examined. However, it was stated that 410 new molecules were accepted by the FDA between 2011 and 2020 (Wang et al., 2022). Our results showed that population growth and cancer medicine consumption have a positive relationship, naturally. However, increased cancer incidence showed a negative relationship with medicine sales. There could be many reasons for this negative relationship. Previously, it was reported in the Turkey Cancer Control Program 2013–2018 that breast cancer, cervical cancer, and colorectal cancer could be diagnosed at a late stage when they become symptomatic in Türkiye (Turkey Cancer Control Programme, 2013-2018, 2016). It was indicated that a late-stage diagnosis may lead to decreased treatment options and expensive treatment needs (McGarvey et al., 2022). Our results are consistent with this literature information.

Original medicines show a positive correlation with population growth, whereas the consumption of generic medicines shows a negative correlation. However, it should not be overlooked that both generic and original medicines show a negative relationship with the exchange rate difference, and the exchange rate difference over the years is observed as a factor that reduces medicines access in Türkiye between 2010 and 2017. Rémuzat et al. reported that the external reference pricing system is widely used in European countries and the spill-over effect of the external reference pricing system affects the market access strategies of pharmaceutical companies (Rémuzat et al., 2015). It has also been shown that external reference pricing strategies lead to delays in the launch time of new medicines (Voehler et al., 2023). In addition, in Türkiye, different mechanisms have been developed to avoid price-related medicine access problems under universal coverage, such as “Medicines Brought from Abroad” for only patients with limited treatment options. However, this pathway of medicines accounted for only 7.5% of total public pharmaceutical expenditure between 2011 and 2017 (Atikeler et al., 2020).

ATC levels 3rd and 4th allow medicines to be classified into chemical, pharmacological, and therapeutic subgroups (WHO Collaborating Centre for Drug Statistics Methodology, 2024). In our study, we classified medicines according to the 3^rd^ level and observed that each group showed different relationships with the examined parameters. In our study, when the relationship between the exchange rate difference and ATC groups was evaluated, the consumption of basic oncology medicines such as antibiotic-based, plant-based, and platinum groups increased with a positive relationship. However, in innovative medicine groups such as monoclonal antibodies and protein kinase inhibitors, consumption shows a negative relationship. As we mentioned before, similar to our results, Voehler et al. showed that external reference pricing strategies lead to delays in new medicines’ launch times (Voehler et al., 2023). High-income countries such as Canada, England, and Germany also used cost containment mechanisms such as price control, and budget caps to control healthcare costs. However, these countries switched their policies to using value-based pricing through health technology assessment and increasing patient co-payment mainly to avoid obstacles to medicine access (Stabile et al., 2013).

An interesting finding in our study was that non-small-cell lung cancer treatment box sales showed a positive relationship with the exchange rate difference; however, it was shown that other diagnoses were negatively related (Table 4). Tracheal, bronchial, and lung cancers are the most frequent cancer types in men, whereas they rank fourth in women in Türkiye. In addition, non-small-cell lung cancer is diagnosed in 79.6% of lung cancer cases (Turkiye Kanser Istatistikleri Yilligi, 2021). Yurdakul et al. reported that 53.9% of lung cancer patients are treated with chemotherapy, except for radiotherapy and surgery options in Türkiye (Yurdakul et al., 2015). However, access to innovative treatments should be evaluated separately. Indeed, Büssgen and Stargardt reported that the availability of new medications has decreased over time from 2000 to 2017 in Türkiye (Büssgen and Stargardt, 2022). Market authorization holders develop strategies for optimal pricing between the countries. So, innovative medicines’ launch can be faster in countries with big pharmaceutical markets like the Netherlands, Sweden, and Germany in Europe (Büssgen and Stargardt, 2022).

With this study, we provide an overview of the cancer medicine access trend in Türkiye. According to the results, pricing policy may be a significant barrier to accessing cancer medicines. Türkiye’s national health coverage provides patients access to medicines with great coverage. Our solution suggests increasing access to cancer medicine first by avoiding using the exchange rate as a cost containment tool and then by applying value-based pricing strategies for innovative medicines. Implementation of these steps over time will minimize the market barriers to oncology medicines.

## Data Availability

The datasets presented in this article are not readily available because box sales data were obtained from IQVIA Turkiye. The use of data is permitted only for this scientific study. Requests to access the datasets should be directed to elif.vural@lokmanhekim.edu.tr.
